# A statistical framework for quantifying clinical equipoise for individual cases during randomized controlled surgical trials

**DOI:** 10.1186/1745-6215-12-258

**Published:** 2011-12-13

**Authors:** Nicholas R Parsons, Yuri Kulikov, Alan Girling, Damian Griffin

**Affiliations:** 1 Warwick Medical School, University of Warwick, Coventry, CV4 7AL, UK; 2 Department of Public Health, Epidemiology and Biostatistics, University of Birmingham, Birmingham, B15 2TT, UK

**Keywords:** Equipoise, Randomised controlled trial, Surgery, Statistical model

## Abstract

**Background:**

Randomised controlled trials are being increasingly used to evaluate new surgical interventions. There are a number of problematic methodological issues specific to surgical trials, the most important being identifying whether patients are eligible for recruitment into the trial. This is in part due to the diversity in practice patterns across institutions and the enormous range of available interventions that often leads to a low level of agreement between clinicians about both the value and the appropriate choice of intervention. We argue that a clinician should offer patients the option of recruitment into a trial, even if the clinician is not individually in a position of equipoise, if there is collective (clinical) equipoise amongst the wider clinical community about the effectiveness of a proposed intervention (the clinical equipoise principle). We show how this process can work using data collected from an ongoing trial of a surgical intervention.

**Results:**

We describe a statistical framework for the assessment of uncertainty prior to patient recruitment to a clinical trial using a panel of expert clinical assessors and techniques for eliciting, pooling and modelling of expert opinions. The methodology is illustrated using example data from the UK Heel Fracture Trial. The statistical modelling provided results that were clear and simple to present to clinicians and showed how decisions regarding recruitment were influenced by both the collective opinion of the expert panel and the type of decision rule selected.

**Conclusions:**

The statistical framework presented has potential to identify eligible patients and assist in the simplification of eligibility criteria which might encourage greater participation in clinical trials evaluating surgical interventions.

## 1 Background

There is an increasing demand for randomised controlled trials (RCTs) in surgery to provide high quality evaluation of new interventions; we use the word intervention synonymously with treatment, procedure or surgical procedure. In a background of ever evolving and improving healthcare, differences between interventions for the same condition are often small, substantially increasing the risk of biased estimation of treatment effects in simple (uncontrolled) observational studies of the interventions [1]. The need for the kind of high level evidence provided by RCTs for surgical interventions is clear [2], although a number of methodological issues have been raised for surgical trials [1,3]. One of the most important issues being recruitment, and specifically identifying whether patients are eligible for entry into a trial.

The existing tremendous diversity in practice patterns across institutions coupled with an ever increasing range of available interventions suggests a low level of agreement between clinicians about both the value of many interventions and the appropriate choice of intervention [4]. A present or imminent controversy in the expert medical community about a choice between interventions is called clinical (or collective) equipoise. Equipoise is the point where we are equally poised in our beliefs about the potential benefits of a particular intervention [5]; i.e. is intervention A better than intervention B. Clinical equipoise is present "if there is genuine uncertainty within the expert medical community - not necessarily on the part of the individual investigator - about the preferred treatment" [5]. In many cases the only way to resolve collective uncertainty about the optimum intervention choice is to undertake a clinical trial. Individual equipoise relates to a single clinician, i.e. the position where he or she has no preference amongst a range of available treatments. It is subject to change for a host of reasons, including peer pressure, the results of potentially imperfect studies and the influence of advertising. Freedman [5] argues that global clinical equipoise should override the individual clinician's lack of equipoise. Clinicians should subsume their personal views and recruit patients into a trial, even if not individually in a position of equipoise themselves. This view is implicitly accepted by society in the form of ethics committees, which must ensure that the treatments being compared are reasonable options before trial participants are sought. Often, for a treatment that is not completely novel, this is demonstrated by the presence of clinical equipoise in an expert and/or wider medical community. Once ethics committee permission has been granted, it then becomes an individual clinician's decision whether the offer of entry into the trial is appropriate for an individual patient [6]. Unfortunately, the varied preferences expressed (which may be rational, anecdotal or irrational) between individual institutions and between individual surgeons within and between institutions often make patient recruitment to trials very challenging.

Statistically the level of individual uncertainty about the effectiveness of an intervention can be quantified by a (subjective) probability, which is assigned to a specific hypothesis and is personal and varies with an individual's knowledge and expertise. "A measure of a state of knowledge" [7] is provided by the Bayesian concept of subjective probability. The process of expert evaluation about the effectiveness of a proposed intervention in an RCT is synonymous with elicitation of a Bayesian prior; i.e. a statement of knowledge prior to performing an experiment or trial usually stated in the form of a probability density. There are a number of approaches to turning informally expressed ideas into a mathematical prior distribution, with no consensus as to the optimal method of determination for a process that is usually problem specific [8]. We choose to elicit the subjective opinion of a panel of experts as a basis for decision making regarding the eligibility of a patient for recruitment to an RCT [9]. This has the advantage of being dynamic and flexible, in the sense that it is quite feasible that opinions will change during the course of a trial, for example with the publication of related research [8], or as experience accumulates amongst clinicians as to how best to undertake a surgical procedure.

Methods for formal measurement of clinical uncertainty, as a prelude to a clinical trial have been suggested previously [10] and measures of surgeon's equipoise in the setting of surgical trials have also been reported [11]. However, we develop these ideas further, using techniques for eliciting subjective judgements before a trial [12-14] and introduce a novel framework for decision making regarding recruitment to an RCT that we hope will be easily understood by clinicians and implemented in real time during the course of a trial. It is particularly challenging recruiting patients to trials comparing operative to non-operative treatments or a standard against a new but popular well-marketed treatment. Therefore we develop a statistical framework to model clinical equipoise (Section 3), using a parametric and a nonparametric approach, for data collected from a clinical trial comparing conservative and operative treatment for displaced fractures of the calcaneus. The results of applying the models are reported in Sections 3.7-10 and we draw conclusions in Section 4.

## 2 Methods

Using available web design tools a method was developed to capture the opinions of clinicians in real time for individual patients (cases) in an ongoing RCT. It comprised of a virtual expert panel giving their opinion about the effectiveness of a proposed treatment for individual patients based on online clinical details; the individual assessments were then synthesized and fed back electronically to the lead clinical investigator. This process is described in greater detail below.

Patients who met the initial trial inclusion criteria were identified and approached by a member of the research team to alert them to the possibility of participating in a trial. They were then asked permission for their anonymized clinical details to be distributed among a panel of experts/clinicians for an opinion regarding the effectiveness of the proposed treatment. Clinical data from consented patients were made available on a secure website managed by eLab at the University of Warwick, and all panel experts/clinicians were alerted by email and text message (if requested) to the posting of a new patient and asked to offer their personal opinion on the likely success of the proposed treatments. The assessment scale is described in more detail for the specific example of the UK Heel Fracture Trial. Initially the system was tested in a pilot study with seven surgeons from five UK hospitals. Ten retrospective calcaneal fracture cases were selected to represent typical variability. The surgeons followed the instructions on the website with online and telephone technical support available; no specific training was given. When voting on all ten cases was completed, surgeons were asked to fill in an evaluation questionnaire. Voting on a single case never took longer than 5 minutes and the available clinical information was found sufficient and the whole process user friendly by all participating surgeons.

After the successful pilot study the system was introduced as an independent component of the UK Heel Fracture Trial, which compared conservative and operative treatment for displaced fractures of the calcaneus. The study had separate ethical approval and a consent form, in addition to the main trial. This allowed inclusion both of those patients who took part in the UK Heel Fracture Trial and those who declined, as soon as the patient met the trial eligibility criteria. To avoid interference with the clinical course, patients were asked permission to use their data at the 6 weeks follow-up clinic or later. Their anonymous clinical data including X-rays and CT images were posted to a secure website. The expert assessment panel included 12 surgeons from 9 hospitals. All surgeons were foot and ankle specialists and acted as principal investigators in their individual trial centres.

After assessing the clinical data available for a given patient, the surgeon was able to scroll down to an interactive scale, featuring bars (initially set at zero) above each of seven outcome categories indicating whether after surgical intervention the patient would get "*much worse*" (1), "*significantly worse*" (2), "*a bit worse*" (3), "*no difference*" (4), "*a bit better*" (5), "*significantly better*" (6) or "*much better*" (7). A left-click of the mouse and a drag allowed each outcome prognosis bar to be set to a desired percentage, which was reported numerically over the bar. Once the assessment summed to 100% (reflected in a digital window in the upper left corner of the scale) the submit button allowed the data to be sent to the trial lead for analysis. The UK Heel Fracture Trial compared operative (surgical) and non-operative (conservative) treatment. Surgical techniques are becoming widespread for calcaneal fracture, but do have associated risks, therefore it was important for the clinician to assess the improvement potential relative to the risks for this procedure. Belief, in the context we describe here, that surgery can make a patient better implies intention to do surgery, while disbelief implies intention to avoid surgical intervention, hence to choose the conservative option. The question posed to the expert panel can and should be tailored to the specific trial. For the UK Heel Fracture Trial the experts were asked to compare operative (surgical) and non-operative (conservative) treatment, which although strongly contrasting treatment options may vary in the exact detail of the constituent components. For studies with less contrasting treatment options (e.g. two types of surgery) the question to experts may simply be whether the test intervention would be better or worse for a patient, compared to a control (standard) intervention.

Table 1 shows four examples of data elicited from between 4 and 6 clinical experts, not necessarily the same individuals labelled as 1 to 6, who provided their opinions on the effectiveness of surgical compared to non-surgical intervention after fracture of the calcaneus. As expected there are clear differences in the both the locations and shapes of the individual distributions for a number of these cases and indeed a number of clear similarities for other cases. For instance, the opinions of the clinicians vary widely for case 1; clinical expert 3 is reasonably confident that the patient will improve significantly after treatment whereas for expert 4 the most likely outcome of treatment is that the condition of the patient will be unchanged. There is much clearer agreement for case 4 where all the experts expect the patient to worsen significantly after treatment. How do we use these data to decide whether a patient (case) is eligible for recruitment to a clinical trial? We propose two approaches here to model the opinions obtained from each expert clinician, a parametric model based on a Beta distribution (Section 3.2) and a nonparametric model based on estimated means and standard deviations (Section 3.3) that characterise expert opinions using concepts of *belief, disbelief *and *uncertainty*. The *belief, disbelief *and *uncertainty *are visualized using a ternary plot that displays these characteristics in a manner that allows them to be compared to decision rules that partition the opinion space. Finally, resampling methods are used to draw inferences concerning the sufficiency of evidence from the clinical experts to patient eligibility for recruitment

**Table 1:** Assessment of the likely effectiveness of surgical intervention after fracture of the calcaneus for four example cases and up to six clinical experts

Case	Assessment	Clinical Expert					
		
		1	2	3	4	5	6
Case 1	Much Worse	5	5	0	0	0	0
	Significantly Worse	5	5	0	0	5	9
	A Bit Worse	10	25	5	15	10	21
	No Difference	20	50	5	59	30	36
	A Bit Better	30	15	15	25	45	23
	Significantly Better	20	0	70	1	10	11
	Much Better	10	0	5	0	0	0

Case 2	Much Worse	0	0	0	0	0	-
	Significantly Worse	0	0	2	0	0	-
	A Bit Worse	10	0	4	10	5	-
	No Difference	15	10	12	13	20	-
	A Bit Better	40	40	32	35	45	-
	Significantly Better	30	50	48	40	30	-
	Much Better	5	0	2	2	0	-

Case 3	Much Worse	10	10	5	5	-	-
	Significantly Worse	10	20	10	15	-	-
	A Bit Worse	15	30	10	20	-	-
	No Difference	20	20	15	20	-	-
	A Bit Better	20	10	30	20	-	-
	Significantly Better	15	10	20	15	-	-
	Much Better	10	0	10	5	-	-

Case 4	Much Worse	20	5	40	10	20	-
	Significantly Worse	60	85	50	80	70	-
	A Bit Worse	15	10	10	5	5	-
	No Difference	5	0	0	5	5	-
	A Bit Better	0	0	0	0	0	-
	Significantly Better	0	0	0	0	0	-
	Much Better	0	0	0	0	0	-

## 3 Results and Discussion

### 3.1 Expert opinion

An opinion regarding the effectiveness of a procedure can be thought of as comprising of three distinctive aspects; *belief, disbelief *and *uncertainty*. *Belief *represents the tendency of an expert to expect a particular treatment to perform better than an alternative (control intervention) for a particular patient (case); i.e. the tendency for the experts to score cases in the higher end categories of the rating scale of Table 1. Conversely, the level of *disbelief *is equated with the tendency for an intervention to have a worse outcome as compared to a control intervention; i.e. the tendency for the experts to score cases in the lower end categories of the rating scale. The *uncertainty *associated with the *belief *and *disbelief *represents the spread of the data across the opinion range; i.e. all the scores might be concentrated in the central category (no difference) or be spread equally between all categories in Table 1 - we would have equal *belief *in these two scenarios but a maximum difference in *uncertainty*.

Borrowing from the notation of subjective logic [15,16], we label the *belief, disbelief *and *uncertainty *associated with an opinion for expert *i *as *b_i_, d_i _*and *u_i_*, and apply the constraint that

(1) $$b_{i}+d_{i}+u_{i}=1\;\text{and}\;\left\{b_{i},d_{i},u_{i}\right\}\in {\left[0,1\right]}^{3}$$

where the triplet π*_i _*= {*b_i_, d_i_, u_i_*} is described as the opinion of expert *i*. Intuitively it makes sense that there should be a constraint on these characteristics, as expressed in (1), as clearly when we have a maximum level of *belief *in a procedure we must necessarily have zero *disbelief *and *uncertainty*. Similarly, when there is a maximum level of *uncertainty *there clearly must be zero levels of *belief *and *disbelief*. The constraint that our levels of *belief, disbelief *and *uncertainty *must sum to unity is of course a matter of convenience, in an analogous manner to that in conventional probability where the same constraint is used. It seems reasonable, using statistical arguments, that we should scale our levels of *belief *and *disbelief *about the effectiveness of a procedure by the associated *uncertainty*. That is we are interested in the quantities *b*/*u *and *d*/*u*, in the same way we might want to normalize a treatment difference in an RCT by the associated standard deviation measuring the spread or uncertainty in the estimated difference to give an effect size. In order to estimate *b, d *and *u*, we need to develop a model for the clinical expert assessment data.

### 3.2 Parametric model

#### 3.2.1 Assessment pooling

The assessment of the likely effectiveness of the intervention *x *was scored on a discrete valued symmetric scale with descriptive terms selected to imply an even spacing between categories. For our selected example, the seven-category ordinal scale, described in Section 2, was transformed onto the interval [0,1] as follows; $1\to \frac{1}{14}$, $2\to \frac{3}{14}$, $3\to \frac{5}{14}$, $4\to \frac{7}{14}$, $5\to \frac{9}{14}$, $6\to \frac{11}{14}$ and $7\to \frac{13}{14}$. This retains the implicit spacing of the ordinal scale and centres the new scale at the same point as the original scale. Equivalent arguments can be constructed for ordinal scales with different numbers of categories.

**Figure 1:**
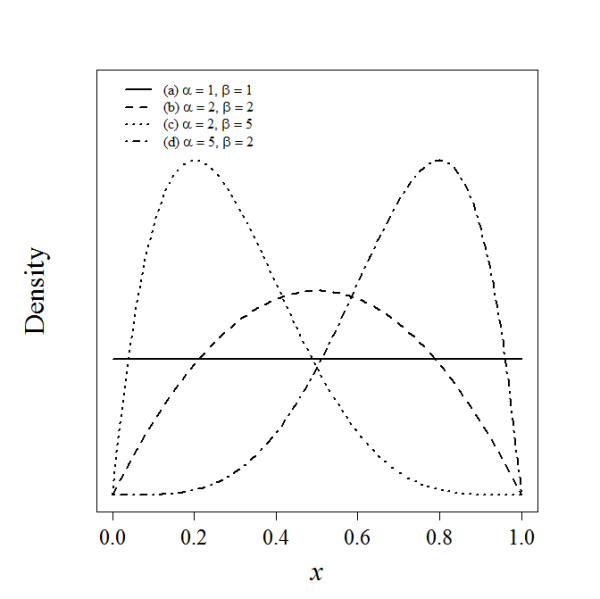
**Beta distributions B(*α, β*) for various values of parameters α and β**.

Let *x_i_*, where 0 ≤ *x_i _*≤1, quantify the likely effectiveness of a procedure for individual expert *i *as part of a panel of *n *experts. The distribution of *x_i _*is assumed to follow an approximate Beta distribution (Figure 1), a continuous probability distribution defined on the interval (0,1) and parameterized by two positive parameters, denoted by α and β, that modify the shape of the distribution. The Beta distribution is widely used for modelling random probabilities, particularly in the context of Bayesian analysis [17] and has been used to describe not only variability within a population as in a conventional statistical model, but also to describe the subjective degree of *belief *in a Bayesian sense [8]. Expressed mathematically, the probability density function for *x_i _*is

$f_{i}\left(x_{i};\alpha_{i},\beta_{i}\right)=\frac{\Gamma \left(\alpha_{i}+\beta_{i}\right)}{\Gamma \left(\alpha_{i}\right)\Gamma \left(\beta_{i}\right)}{x_{i}}^{\alpha_{i}-1}{\left(1-x_{i}\right)}^{\beta_{i}-1},$,

where Γ(.) is the gamma function and parameters *α_i _*≥ 1 and *β_i _*≥ 1, requiring that the distribution be unimodal or at the extreme case, when *α_i _*= *β_I _*= 1, uniform. In the surgical trial setting described here, it seems unlikely that for instance a u-shaped distribution for *x_i _*(e.g. *α *= 0.5 and *β *= 0.5) would be plausible.

The multiplicative pooled assessment [18,19] of the expert panel is obtained as

$$f_{0}\left(x\right)={\left\{\prod_{i=1}^{n}f_{i}\left(x_{i};\alpha_{i},\beta_{i}\right)\right\}}^{1∕n}$$

where *f_0_*(*x*) follows a Beta distribution with parameters $\bar{\alpha }=\frac{1}{n}\sum_{i=1}^{n}\alpha_{i}$ and $\bar{\beta }=\frac{1}{n}\sum_{i=1}^{n}\beta_{i}$. This provides a pooled assessment that represents the intersection of the beliefs of the expert panel [19].

#### 3.2.2 Opinion model

In order to translate the assessments from the panel of *n *experts to a collective expert opinion, the measures $\bar{b}/\bar{u}$ and $\bar{d}/\bar{u}$ (Section 3.1), that characterise the pooled opinion, are related to the pooled assessment parameters $\bar{\alpha }$ and $\bar{\beta }$. Equating the level of belief expressed by an expert to the pooled assessments, it is clear that $\bar{\alpha }$ must be proportional to $\bar{b}/\bar{u}$, that is a larger value of $\bar{\alpha }$ represents a greater degree of *belief*; at the extreme as $\bar{\alpha }\to \infty$, then $\bar{b}\to 1$ and ū→0, when we have maximum *belief *we must have minimum *uncertainty*. Similarly arguments lead to $\bar{\beta }$ being proportional to $\bar{d}/\bar{u}$; a larger value of $\bar{\beta }$ represents a greater degree of *disbelief*. Although, clearly from example (a) in Figure 1, when the pooled Beta distribution parameter estimates are at their minimum and $\bar{\alpha }=1$ and $\bar{\beta }=1$ then there is maximum *uncertainty *(ū=1) and minimum *belief *and *disbelief*, $\bar{b}=\bar{d}=0$. Formalizing these arguments leads to the following expressions that satisfy all these conditions

(2-3) $$\frac{\bar{b}}{ū}=\bar{\alpha }-1\;\text{and}\;\frac{\bar{d}}{ū}=\bar{\beta }-1.$$

Solving equations (2) and (3), along with the condition that $\bar{b}+\bar{d}+ū=1$ (equation 1), yields the following expressions that characterize the relationship between the triplet $\left\{\bar{b},\bar{d},ū\right\}$ and the parameters $\bar{\alpha }$ and $\bar{\beta }$,

(4-6) $$\bar{b}=\frac{\bar{\alpha }-1}{\bar{\alpha }+\bar{\beta }-1},\;\bar{d}=\frac{\bar{\beta }-1}{\bar{\alpha }+\bar{\beta }-1},\;\text{and}\;ū=\frac{1}{\bar{\alpha }+\bar{\beta }-1};$$

where the triplet $\left\{\bar{b},\bar{d},ū\right\}$ clearly satisfies $\bar{b}+\bar{d}+ū=1$; a more detailed derivation of equations (4)-(6) is provided elsewhere [15,16]. Thus, when $\bar{\alpha }=\bar{\beta }=1$, $\bar{\pi }=\left\{0,0,1\right\}$ and the pooled opinion is total *uncertainty *(ignorance); see example (a) in Figure 1. If parameters $\bar{\alpha }$ and $\bar{\beta }$ are greater than unity but equal, we have equal *belief *and *disbelief*; for example (b) in Figure 1 where $\bar{\alpha }=\bar{\beta }=2$ and $\bar{\pi }=\left\{\frac{1}{3},\frac{1}{3},\frac{1}{3}\right\}$. As $\bar{\alpha }$ increases relative to $\bar{\beta }$ the *belief *increases and the *uncertainty *decreases and conversely as $\bar{\beta }$ increases relative to $\bar{\alpha }$ the *disbelief *increases and the *uncertainty *decreases; these two scenarios are illustrated in examples (d) and (c) in Figure 1, where $\bar{\alpha }=5$, $\bar{\beta }=2$ and $\bar{\pi }=\left\{\frac{4}{6},\frac{1}{6},\frac{1}{6}\right\}$ and $\bar{\alpha }=2$, $\bar{\beta }=5$ and $\bar{\pi }=\left\{\frac{1}{6},\frac{4}{6},\frac{1}{6}\right\}$.

### 3.3 Nonparametric model

An alternative nonparametric formulation for *belief, disbelief *and *uncertainty *allows a more general approach to that described in Section 3.2. Defining *μ_i _*and σ*_i _*as the mean and standard deviation of the assessment of the effectiveness of the intervention *x_i _*for expert *i*, where *x_i _*is in the range [0,1]. Then the *uncertainty *(*u_i_*), *belief *(*b_i_*) and *disbelief *(*d_i_*) associated with an opinion for expert *i *can be expressed as $u_{i}=\sigma_{i}^{2}/\mu_{i}(1-\mu_{i})$, *b_i _*= *μ_i_*(1-*u_i_*) and *b_i _*= *d_i _*=(1-*μ_i_*)(1-*u_i_*); as 0 ≤ *x_i _*≤ 1, then 0 ≤ *u_i _*≤ 1 and the measures satisfy equation (1). For example using the data from Table 1 for expert 4 from case 3, the weighted mean and standard deviation, based on the transformed seven-category ordinal scale described in Section 3.2.1 (1∕14,3∕14,5∕14,7∕14,9∕14,11∕14,13∕14) with weights given by (5,15,20,20,20,15,5), are *μ *= 0.5 and σ = 0.226, and so *u *= 0.204 and *b *= *d *= 0.398. Multiplicative pooling leads directly to estimates for the opinion triplet $\left\{\bar{b},\bar{d},ū\right\}$, with weights given by the *n*^th ^root of the product of the individual expert weights, in an analogous manner to that described in Section 3.2.1 for the parametric model.

In fact the expressions for *uncertainty, belief *and *disbelief *for the Beta model in equations (4)-(6) follow directly from the above expressions for *u, b *and *d*, based on *μ *and σ, after some rescaling, by noting that the mean and variance of the Beta distribution are *α*/(*α*+*β*) and *αβ*/{(*α*+*β*)^2 ^(*α*+*β*+1)} respectively.

### 3.4 Opinion space

As proposed by Jøsang [15], a ternary plot provides a convenient method of representing the triplet of *belief, disbelief *and *uncertainty *that constitute a pooled expert opinion. A ternary plot represents the ratios of the three variables as positions in an equilateral triangle, where each base, or side, of the triangle represents a proportion, with the point of the triangle opposite that base representing a proportion equal to one. As a proportion increases in any one sample, the point representing that sample moves from the base to the opposite point of the triangle. For instance, when $\bar{\alpha }=\bar{\beta }=1$ (maximum *uncertainty*) the opinion is mapped to the apex of the equilateral triangle, whereas when $\bar{\alpha }=\bar{\beta }=2$ there is equal *belief, disbelief *and *uncertainty *and the pooled opinion is mapped to the centre of the triangle. The cases representing greater levels of *belief *and greater levels of *disbelief *are mapped towards the right-hand and left-hand vertices of the triangle respectively.

### 3.5 Decision rules

In order to determine the level of equipoise that should be satisfied for a clinical trial to be considered ethical Johnson *et al*. [20] conducted an ethometric study to investigate how much clinical equipoise can be disturbed before potential trial subjects deem it to be unethical. A series of hypothetical clinical trial scenarios were presented to people from a broad range of societal and geographical groups within the UK. They were asked to specify the level of collective doubt between two treatment modalities that they would accept if casting a vote on an ethics committee. Johnson *et al*. [20] defined the 80:20 rule, that represented the split in equipoise that should be allowed for a trial to be judged to be ethical and recommended its use as an appropriate tool for deciding whether recruitment is ethically justifiable; based on their empirical evidence that less than 3% of subjects questioned thought that a trial should morally be undertaken if equipoise was beyond this point. By way of comparison, an alternative *mean threshold *rule might consider it ethical to recruit patients if the mean clinical effectiveness (*μ*), estimated as *α*/(*α*+*β*) for the Beta distribution, were within pre-determined limits. For instance, it might be considered ethical to recruit patients into a trial if the mean clinical effectiveness were in the range 0.4 ≤ *μ *≤ 0.7.

The 80:20 and *mean threshold *equipoise decision rules can be mapped onto the opinion space and visualized on a ternary plot. For the Beta model (Section 3.2), the former rule can be mapped on to the ternary plot by iteratively finding solutions for Beta distribution parameters, α and β, that give estimates for the probability density function equal to 0.2 and 0.8 to the left and right of the central point on the expert rating scale, and for the latter rule by simply solving equations (4)-(6) using the constraint that *μ*(*α*+*β*)=*α*.

### 3.6 Hypothesis testing

The significance of the estimated pooled opinion ($\bar{\pi }$) is assessed using resampling. For the Beta model for Section 3.2, pooled assessment parameters ${{\bar{\alpha }}_{m}}^{*}=\frac{1}{n}\sum_{i^{*}\in S_{m}}\alpha_{i^{*}}$ and ${{\bar{\beta }}_{m}}^{*}=\frac{1}{n}\sum_{i^{*}\in S_{m}}\beta_{i^{*}}$ are estimated for *S_m_*, a set of size *n *constructed by sampling with replacement from {1,2...*n*}; for example for the pooled assessment of 5 experts *S_m _*might be {1,2,2,4,1} or {5,3,3,1,1}. This process is repeated many times by random construction of *S_m _*to give empirical bootstrap [21] distributions ${{\bar{\alpha }}_{1}}^{*},{{\bar{\alpha }}_{2}}^{*},\ldots ,{{\bar{\alpha }}_{M}}^{*}$ and ${{\bar{\beta }}_{1}}^{*},{{\bar{\beta }}_{2}}^{*},\ldots ,{{\bar{\beta }}_{M}}^{*}$, and thereby ${{\bar{\pi }}_{1}}^{*},{{\bar{\pi }}_{2}}^{*},\ldots ,{{\bar{\pi }}_{M}}^{*}$. From this empirical distribution, a bootstrap confidence interval for $\bar{\pi }$ is derived for the purpose of hypothesis testing. A similar resampling scheme can also be developed simply for the nonparametric model of Section 3.3.

This resampling methodology represents the variability in opinion that might be obtained for any combination of experts in the panel, including in principle a panel composed entirely of a single expert, and as such represents the full range of possible opinions for the selected population of experts. For the relative small panel of experts in our example, exhaustive permutation resampling [21] is the preferred option, but this may be computational unrealistic for large *n *where bootstrapping with *M *= 1000 would be sufficient.

### 3.7 Beta distribution fitting

The outlined statistical framework is illustrated using the example data introduced in Section 2 (Table 1). We focus here on the Beta model (Section 3.2) as an exemplar, as this fits our data well and is computational slightly more complex to implement than the nonparametric method. Statistical analysis was undertaken in the statistical software package R [22]. Code to replicate the analysis presented here is available on request from the corresponding author.

**Figure 2:**
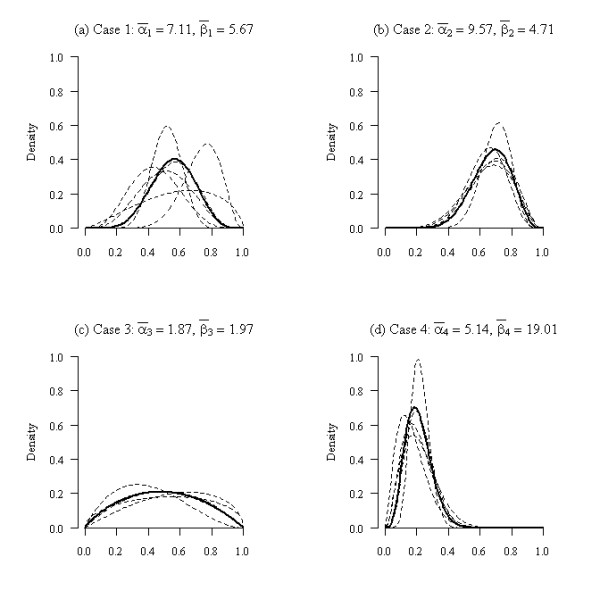
**Fitted Beta distributions for each clinical expert (---) and pooled estimates (**-**) for each case**.

The parameters of the Beta distribution were estimated for each clinical expert for the four cases shown in Table 1 using the fitdistr function available in the MASS [23] library in the statistical software package R [22]. This function estimates parameters for a range of univariate distributions, including the Beta distribution, using maximum-likelihood methods. For the four example cases introduced in Section 2 the pooled parameter estimates were ${\bar{\alpha }}_{1}=7.11$, ${\bar{\alpha }}_{2}=9.57$, ${\bar{\alpha }}_{3}=1.87$, ${\bar{\alpha }}_{4}=5.14$ and ${\bar{\beta }}_{1}=5.67$, ${\bar{\beta }}_{2}=4.71$, ${\bar{\beta }}_{3}=1.97$, ${\bar{\beta }}_{4}=19.01$. The fitted distributions for each clinical expert and pooled estimates are shown in Figure 2.

### 3.8 Opinions

The pooled parameter estimates from the Beta distribution fitting for the four example cases were used to estimates the *belief, disbelief *and *uncertainty *using equations (4)-(6); this gave the following estimates, ${\bar{b}}_{1}=0.518\;$, ${\bar{b}}_{2}=0.645\;$, ${\bar{b}}_{3}=0.307$, ${\bar{b}}_{4}=0.179\;$, ${\bar{d}}_{1}=0.397\;$, ${\bar{d}}_{2}=0.279\;$, ${\bar{d}}_{3}=0.341\;$, ${\bar{d}}_{4}=0.778\;$ and ${ū}_{1}=0.085\;$, ${ū}_{2}=0.075\;$, ${ū}_{3}=0.351\;$, ${ū}_{4}=0.043\;$. Inspection of Figure 2, indicates that there appears to be significant belief for case 2 that the patient will improve after treatment (surgery) and conversely significant disbelief in the effectiveness of the treatment for case for case 4; this is reflected in the large (> 0.6) estimates of *b *and *d *for cases 2 and 4 respectively. Also, there is significant uncertainty, seen by the flatness of the curves in Figure 2(c), in the collective opinions of the experts for case 3; this is apparent in the large level of uncertainty for this case, relative to the other cases.

### 3.9 Decision rules

In order to determine whether an opinion provides sufficient evidence for eligibility for recruitment to a clinical trial, we must first define a decision rule. Here we focus on two rules, the 80:20 [20] and the *mean threshold *rules; although the procedures described here are equally applicable to many more rules that could potentially be defined. The 80:20 and *mean threshold *rules partition the opinion space, visualized by the ternary plot, into regions that determine whether the patient can or cannot ethically be recruited to a trial.

**Figure 3:**
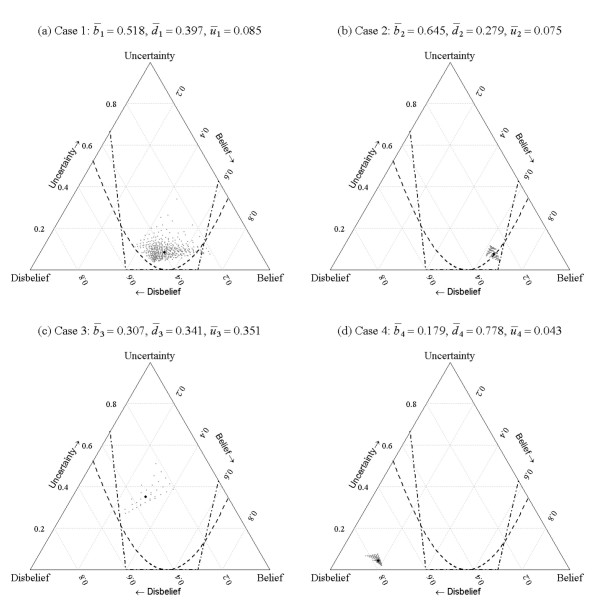
**Estimated triplets for all permutations of opinions with the 80:20 (---) and mean (--) decision rules**. The best estimate of collective opinion is given by the large symbol (•).

The division lines between the regions for the 80:20 rule were determined iteratively (using an interval search method) by finding estimates of the Beta distribution parameters α and β that exactly divided the probability density 80% and 20% around equipoise, and projecting these estimates into the opinion space using equations (4)-(6). This process was achieved using an implementation of the uniroot function in R [22]. After discussion with the clinical experts it became clear that the point of equipoise for the assessment scale described in Section 2 for the 80:20 rule was not located centrally but was in fact located at the division between the '*No difference*' and the '*A bit better*' categories. That is, because surgery was seen to be an active intervention for a condition that required treatment, the point of equipoise was located slightly to the right of the centre point of the assessment scale; which for our definition of the assessment scale is at 8∕14 rather than at 1∕2 on the interval (0, 1). The asymmetry that this implies for the 80:20 decision rule is clear in Figure 3. The *mean threshold *rule divided the opinion space into three distinct regions *μ *< 0.4, 0.4 ≤ *μ *≤ 0.7 and *μ *< 0.7 characterised by the thresholds 0.4 and 0.7 for the mean, that determined whether the intervention was likely to be effective. The divisions between regions were mapped onto the opinion space by solving equations (4)-(6) using the constraint that *μ*(*α*+*β*) = *α*. For instance for *μ *= 0.7 and *u *= 0, then *b *= 0.7 and *d *= 0.3 and when *d *= 0 then u=3∕7 and u=4∕7; these points define the intersections between the upper division boundary with the lower and right edges of the ternary plot in Figure 3.

### 3.10 Hypothesis testing

The exhaustive permutation test described in Section 3.6 was applied to each of the test cases. This gave 462, 126, 35 and 126 combinations of opinions for the four cases that used respectively 6, 5, 4 and 5 expert clinical assessors. The *belief, disbelief *and *uncertainty *for all the combinations of opinion were estimated for each of the four cases and plotted along with the decision rules in Figure 3.

The 'cloud' of points for each case represents the variability due to the range of opinions expressed by the expert assessors. Where there were considerable differences of opinion, for instance for case 1, there was a much wider spread of points than where there was overall agreement amongst the experts about the likelihood of success of the intervention, for instance for case 2 or 4. It is instructive to look at one particular opinion triplet to more fully understand the meaning of the ternary plots.

For case 1, the opinion triplet *π *= {0.712,0.211,0.077} located towards the lower right hand vertex of the ternary plot has very high belief and low uncertainty. This is the opinion associated with six replicates of the assessment of clinical expert 3 for case 1 (see Table 1), who had a strong belief that the patient would get significantly better after treatment. If this expert assessor were indeed representative of the wider population of experts, then it would certainly be unethical for the patient to be recruited to the trial and consequentially the opinion for this potential scenario is located to the right of the 80:20 and *mean threshold *decision rules.

Labelling the regions to the lower right and lower left of the plots to the right and left of the 80:20 and *mean threshold *decision rule partition curves as the 'belief' and 'disbelief' regions, allows us to count the number of opinions falling within these regions for each case and rule; see Table 2. Defining the null hypothesis to be that a case should not be recruited to the trial, Table 2 provides evidence for this hypothesis and suggests appropriate *p*-values based on the 80:20 rule for the four cases to be 0.026 (i.e. 12/462), 0.333, 0.000 and 1.000 and based on the *mean threshold *rule to be 0.011 (i.e. 5/462), 0.000, 0.029 and 1.000. Testing at the 5% level (two-sided) indicates that for the 80:20 rule cases 1 and 3 would be eligible for recruitment and for the *mean threshold *rule cases 1, 2 and 3 would be eligible for recruitment. For this decision making process to have some validity, the decision rule and the significance level would clearly need to be stated before data collection was undertaken.

**Table 2:** Opinion counts by case, decision region and rule, and the total number of opinion combinations available for the exhaustive permutation test

Rule	Region	Case 1	Case 2	Case 3	Case 4
80:20	Belief	12	42	0	0

	Disbelief	0	0	0	126

*Mean threshold*	Belief	5	0	0	0
	Disbelief	0	0	1	126

Opinion Combinations		462	126	35	126

## 4 Conclusions

We describe a statistical framework for the assessment of clinical uncertainty, as a prelude to a clinical trial and demonstrate, using data from the UK Heel Fracture Trial, how expert opinions can be pooled, modelled and presented on a ternary plot that represents an opinion space. Individual cases can then be assessed in relation to decision rules mapped onto the opinion space, providing clear and rapid decisions regarding trial eligibility. The methodology has potential to identify eligible patients and assist in the simplification of eligibility criteria which might encourage greater participation in clinical trials.

Methods for the assessment of clinical uncertainty, as a prelude to a clinical trial, have been suggested previously [10,11]. However, the methodology described here is the first attempt at a structured statistical framework to undertake this type of analysis. Beta distributions were fitted to assessments of the likely effectiveness of an intervention elicited from a virtual panel of experts and pooled using methods familiar to exponents of determining expert probabilities [19]. Opinions were expressed using previously suggested [15] definitions of *belief, disbelief *and *uncertainty *that we believe fully characterised the clinical expert assessments. Our analysis restricted the choice of Beta distributions for modelling to unimodal forms (*α *≥ 1 and *β *≥ 1). This was not a concern for the examples described here or indeed more widely for other data we have explored in the setting of surgical trials. However, it is in principle possible in other applications that the most likely assessment of clinical effectiveness of an intervention is that a patient would either get *much better *or *much worse *with any other outcome being extremely unlikely. In this setting *belief, disbelief *and *uncertainty *as expressed in equations (4)-(6) would not be defined. For the data presented here the Beta model proved to be the most informative, however where this is not the case the nonparametric methods described, based on estimated means and standard deviations, provide useful alternatives for any distribution on the interval [0,1]. Although the examples described here all use seven point likert type scales for elicitation, the statistical framework introduced would work equally well with any type of ordered categorical assessment scale.

Expert opinions are pooled here using multiplicative methods [19], as we felt that this best represented clinical equipoise [24] and the views of the experts consulted for the example data; i.e. that all experts opinions were 'correct' and the pooling should represent the consensus based on the intersection of beliefs. However, our view is pragmatic and we see no reason why additive pooling could not be used in preference to multiplicative pooling, particularly if it was felt that the latter method was giving too much weight to the assessment of one or more 'over-confident' individual experts.

We have presented significance tests at the 5% level to assess whether a patient might ethically be recruited to a trial. Our selection of this level for the tests was somewhat arbitrary and clearly this could be set, prior to analysis, at a higher or lower level for a different application or a less formal procedure adopted if necessary. The 80:20 rule [20], which is based on some empirical evidence, was selected as a standard for decision making regarding recruitment. The alternative *mean threshold *rule, as well as being intuitively reasonable, was suggested in part to encourage some debate as to what form the decision rule should take for different cases and in various settings. This is clearly an area that requires additional research.

The focus of this paper has been on developing tools for improving recruitment to trials. For those patients deemed eligible for recruitment who decide to enter an RCT, it would seem natural to use the expert evidence elicited through this process as a clinical prior, based on subjective opinion, in a formal Bayesian analysis [14].

The methodological framework discussed here has provided additional insight that would otherwise have not been available for the heel fracture trial. Although, clearly this methodology will need to be assessed in future studies to identify whether it can actually deliver improvement in trial recruitment rates. The methodological framework we describe is currently limited to two-arm trials, although we see no reason why this could not be extended to more than two treatment groups. The opinion pooling we describe is appropriate for situations where individual expert opinions may differ to a moderate or large extent, but it is not at all clear that pooling opinions where for instance experts have totally opposing views (100% *belief *or *disbelief *in treatment effectiveness) would be appropriate, as the pooled opinion would in reality represent no individual expert's opinion. Therefore we would recommend the methodology be limited to only those scenarios of the former rather than the latter type. Although we have focussed on surgical trials, we would expect the methodology described here to be applicable to any RCT where recruitment was problematic. The methodology also has clear application in pilot studies where feasibility is being assessed and also potentially as a support tool for inclusive trials where patients are allowed to select an intervention as well as being randomised in a conventional manner [25].

## Competing interests

The authors declare that they have no competing interests.

## Authors' contributions

DG, chief investigator for the UK Heel Fracture Trial, and YK developed the original concepts for the study. NP developed the statistical methodology, with input from YK and AG, and wrote the first draft of the paper. All authors contributed to the paper during development and read and approved the final version of the manuscript.
